# Supplementary material for: Remimazolam for anesthesia and sedation in pediatric ambulatory surgery: A scoping review protocol
Source: PLoS One. 2025 Aug 5;20(8):e0329861. doi: 10.1371/journal.pone.0329861 (PMC12324083; doi:10.1371/journal.pone.0329861)
Supplement: S1 Appendix — (DOCX) [file pone.0329861.s001.docx]

## Appendix A: Preliminary Search Strategy for PubMed Database

( "Remimazolam"[Supplementary Concept] OR remimazolam[tiab] OR "CNS 7056"[tiab] OR "ONO-2745"[tiab] OR "Byfavo"[tiab] OR "Anerem"[tiab] )

AND

("Child"[Mesh] OR "Infant"[Mesh] OR "Adolescent"[Mesh] OR "Pediatrics"[Mesh] OR "Child, Preschool"[Mesh] OR pediatric[tiab] OR child[tiab] OR infant[tiab] OR neonat[tiab] OR adolescen[tiab] OR boy[tiab] OR girl[tiab] OR paediatr[tiab] )

AND

( "Ambulatory Surgical Procedures"[Mesh] OR "Anesthesia, Intravenous"[Mesh] OR "Conscious Sedation"[Mesh] OR "Deep Sedation"[Mesh] OR "Anesthesia, General"[Mesh] OR "ambulatory surgery"[tiab] OR "day surgery"[tiab] OR "outpatient surgery"[tiab] OR "anesthesia"[tiab] OR "sedation"[tiab] OR "procedural sedation"[tiab] OR "day-case surgery"[tiab] OR "office-based anesthesia"[tiab] )
