# Supplementary material for: Remimazolam for anesthesia and sedation in pediatric ambulatory surgery: A scoping review protocol
Source: PLoS One. 2025 Aug 5;20(8):e0329861. doi: 10.1371/journal.pone.0329861 (PMC12324083; doi:10.1371/journal.pone.0329861)
Supplement: S1 Checklist — (DOCX) [file pone.0329861.s002.docx]

**PRISMA-P 2015 Checklist**

# **This checklist has been adapted for use with protocol submissions to *Systematic Reviews* from Table 3 in Moher D et al**:**** Preferred reporting items for systematic review and meta-analysis protocols (PRISMA-P) 2015 statement. *Systematic Reviews* 2015 ****4****:1

| **Section/topic** | **#** | **Checklist item** | **Information reported** | | **Line number(s)** |  |
| --- | --- | --- | --- | --- | --- | --- |
|  |  |  | **Yes** | **No** |  |  |
| **ADMINISTRATIVE INFORMATION** | | | | | | |
| **Title** | | | | | | |
| Identification | 1a | Identify the report as a protocol of a systematic review |  |  | 1-2 (“…A Scoping Review Protocol”) |  |
| Update | 1b | If the protocol is for an update of a previous systematic review, identify as such |  |  | Not stated as an update. 203-204 (“This study protocol outlines a scoping review that has not yet commenced data collection or analysis.”) |  |
| **Registration** | 2 | If registered, provide the name of the registry (e.g., PROSPERO) and registration number in the Abstract |  |  | Registration is not mentioned in the Abstract (18-65) or elsewhere. |  |
| **Authors** | | | | | | |
| Contact | 3a | Provide name, institutional affiliation, and e-mail address of all protocol authors; provide physical mailing address of corresponding author |  |  | 3-10 (Author affiliations); 11-17 (Corresponding Author: Yunbo Mo, Affiliation, Address, Email) |  |
| Contributions | 3b | Describe contributions of protocol authors and identify the guarantor of the review |  |  | 307-318 (Author Contributions section details individual contributions. Yunbo Mo (314) is listed as overseeing the project.) |  |
| **Amendments** | 4 | If the protocol represents an amendment of a previously completed or published protocol, identify as such and list changes; otherwise, state plan for documenting important protocol amendments |  |  | Not stated as an amendment. No explicit plan for documenting future protocol amendments is detailed, though adherence to JBI/PRISMA (38-42, 137-142) implies such practices if amendments occur. |  |
| **Support** | | | | | | |
| Sources | 5a | Indicate sources of financial or other support for the review |  |  | 321-323 (Funding section: “Funding for this study comes from the Wanzhou District Science and Health Joint Initiative of Chongqing City (Project No.: wzwjw-kw2024031).”) |  |
| Sponsor | 5b | Provide name for the review funder and/or sponsor |  |  | 321-323 (Funding section: “Wanzhou District Science and Health Joint Initiative of Chongqing City.”); 328-330 (Acknowledgements also mentions “Wanzhou District Health Commission and the Science and Technology Bureau of Chongqing City.”) |  |
| Role of sponsor/funder | 5c | Describe roles of funder(s), sponsor(s), and/or institution(s), if any, in developing the protocol |  |  | 321-323 (The Funding section mentions financial support.) 328-330 (The Acknowledgements section thanks them for “collaborative financial support” but does not describe a role in developing the protocol.) |  |
| **INTRODUCTION** | | | | | | |
| **Rationale** | 6 | Describe the rationale for the review in the context of what is already known |  |  | 69-107 (Introduction -> Rationale section) |  |
| **Objectives** | 7 | Provide an explicit statement of the question(s) the review will address with reference to participants, interventions, comparators, and outcomes (PICO) |  |  | 28-37 (Abstract -> Objective); 108-135 (Introduction -> Objectives: details the aim (109-115) and essential questions (116-135) which align with PCC for scoping reviews: Population - pediatric groups; Concept - remimazolam use, dosing, efficacy, safety, comparisons, economics; Context - clinical settings, comorbidities) |  |
| **METHODS** | | | | | | |
| **Eligibility criteria** | 8 | Specify the study characteristics (e.g., PICO, study design, setting, time frame) and report characteristics (e.g., years considered, language, publication status) to be used as criteria for eligibility for the review |  |  | 143-171 (Methods -> Inclusion Criteria: Population (146-151), Concept (152-162), Context (163-171) subsections detail these based on PCC framework); 172-183 (Methods -> Study Types); 47-48, 216-217 (timeframe “from the creation of these databases up until March 31, 2025” / “from the beginning of each database up until March 31, 2025”). |  |
| **Information sources** | 9 | Describe all intended information sources (e.g., electronic databases, contact with study authors, trial registers, or other grey literature sources) with planned dates of coverage |  |  | 42-46, 184-201 (Methods -> Information Sources: lists databases (43-46, 186-195), trial registries (197-201), manual searches of reference lists (196-197)). Planned dates of coverage: 47-48, 216-217 (“from the creation of these databases up until March 31, 2025” / “from the beginning of each database up until March 31, 2025.”) |  |
| **Search strategy** | 10 | Present draft of search strategy to be used for at least one electronic database, including planned limits, such that it could be repeated |  |  | 212-218 (Methods -> Search Strategy); Appendix A (referenced on line 214) contains the PubMed strategy. |  |
| ***STUDY RECORDS*** | | | | | | |
| Data management | 11a | Describe the mechanism(s) that will be used to manage records and data throughout the review |  |  | 51-52, 219-221 (Methods -> Literature Screening: “All collected records will be uploaded into reference management tools (such as EndNote or Rayyan QCRI).”) |  |
| Selection process | 11b | State the process that will be used for selecting studies (e.g., two independent reviewers) through each phase of the review (i.e., screening, eligibility, and inclusion in meta-analysis) |  |  | 51-52, 221-231 (Methods -> Literature Screening: “…a two-phase screening procedure will be carried out independently by two reviewers… Disputes…resolved through discussion; if consensus is not reached, a third reviewer will intervene.”) |  |
| Data collection process | 11c | Describe planned method of extracting data from reports (e.g., piloting forms, done independently, in duplicate), any processes for obtaining and confirming data from investigators |  |  | 52-55, 232-239 (Methods -> Data Extraction: “…conducted separately by two reviewers utilizing a standardized data extraction form that has been both pre-designed and pilot-tested…extracted information will undergo a cross-check, and any inconsistencies will be addressed through discussion, with a third reviewer stepping in for arbitration if required…”) |  |
| **Data items** | 12 | List and define all variables for which data will be sought (e.g., PICO items, funding sources), any pre-planned data assumptions and simplifications |  |  | 53-55, 232-254 (Methods -> Data Extraction lists categories of data (241-254)); Table 1 (referenced on line 235, 255-256) details specific data items under categories like Study Characteristics, Participant Characteristics, Intervention Details, Outcome Measures, etc. |  |
| **Outcomes and prioritization** | 13 | List and define all outcomes for which data will be sought, including prioritization of main and additional outcomes, with rationale |  |  | 34-35 (Abstract -> Objective); 116-135 (Introduction -> Objectives (key questions imply priorities)); 54-55, 240-254 (Methods -> Data Extraction lists outcome categories); Table 1 (referenced on line 235, 255-256) details “Efficacy Outcomes,” “Safety Outcomes,” “Patient and Operator Experience,” “PK/PD Parameters,” “Economic Costs”. While not explicitly labelled “main” vs “additional,” the structure implies prioritization. |  |
| **Risk of bias in individual studies** | 14 | Describe anticipated methods for assessing risk of bias of individual studies, including whether this will be done at the outcome or study level, or both; state how this information will be used in data synthesis |  |  | 257-259 (Methods -> Data Synthesis and Presentation of Results: “This scoping review is not intended to include a thorough assessment of bias risk in the selected studies…”); 293-295 (Discussion (Preliminary Expectations): “…it does not include a comprehensive quality assessment of the studies reviewed…”) (States it will not be done). |  |
| ***DATA*** | | | | | | |
| **Synthesis** | 15a | Describe criteria under which study data will be quantitatively synthesized |  |  | 258-259 (Methods -> Data Synthesis and Presentation of Results: “…not intended…to carry out meta-analyses for synthesizing effect estimates.”) (States it will not be done). |  |
|  | 15b | If data are appropriate for quantitative synthesis, describe planned summary measures, methods of handling data, and methods of combining data from studies, including any planned exploration of consistency (e.g., *I* ^2^, Kendall’s tau) |  |  | N/A (as quantitative synthesis is not planned, stated on lines 258-259). |  |
|  | 15c | Describe any proposed additional analyses (e.g., sensitivity or subgroup analyses, meta-regression) |  |  | No such additional analyses are proposed. |  |
|  | 15d | If quantitative synthesis is not appropriate, describe the type of summary planned |  |  | 55-57, 259-262, 274-276 (Methods -> Data Synthesis and Presentation of Results: “…the data will be summarized in a narrative format, enhanced by tables and figures… Quantitative data such as the count of studies, participant numbers, and age ranges will be assessed through descriptive statistical methods like frequencies and percentages.”) |  |
| **Meta-bias(es)** | 16 | Specify any planned assessment of meta-bias(es) (e.g., publication bias across studies, selective reporting within studies) |  |  | No assessment of meta-biases is mentioned. |  |
| **Confidence in cumulative evidence** | 17 | Describe how the strength of the body of evidence will be assessed (e.g., GRADE) |  |  | No assessment of the strength of the body of evidence (like GRADE) is planned. 294-296 (The Discussion (Preliminary Expectations) states: “…preventing the derivation of strong conclusions regarding the actual impacts of different interventions.”) |  |
