# Supplementary figures and images for: Remimazolam for anesthesia and sedation in pediatric ambulatory surgery: A scoping review protocol
Source: PLoS One. 2025 Aug 5;20(8):e0329861. doi: 10.1371/journal.pone.0329861 (PMC12324083; doi:10.1371/journal.pone.0329861)

# PRISMA-ScR Flow Diagram

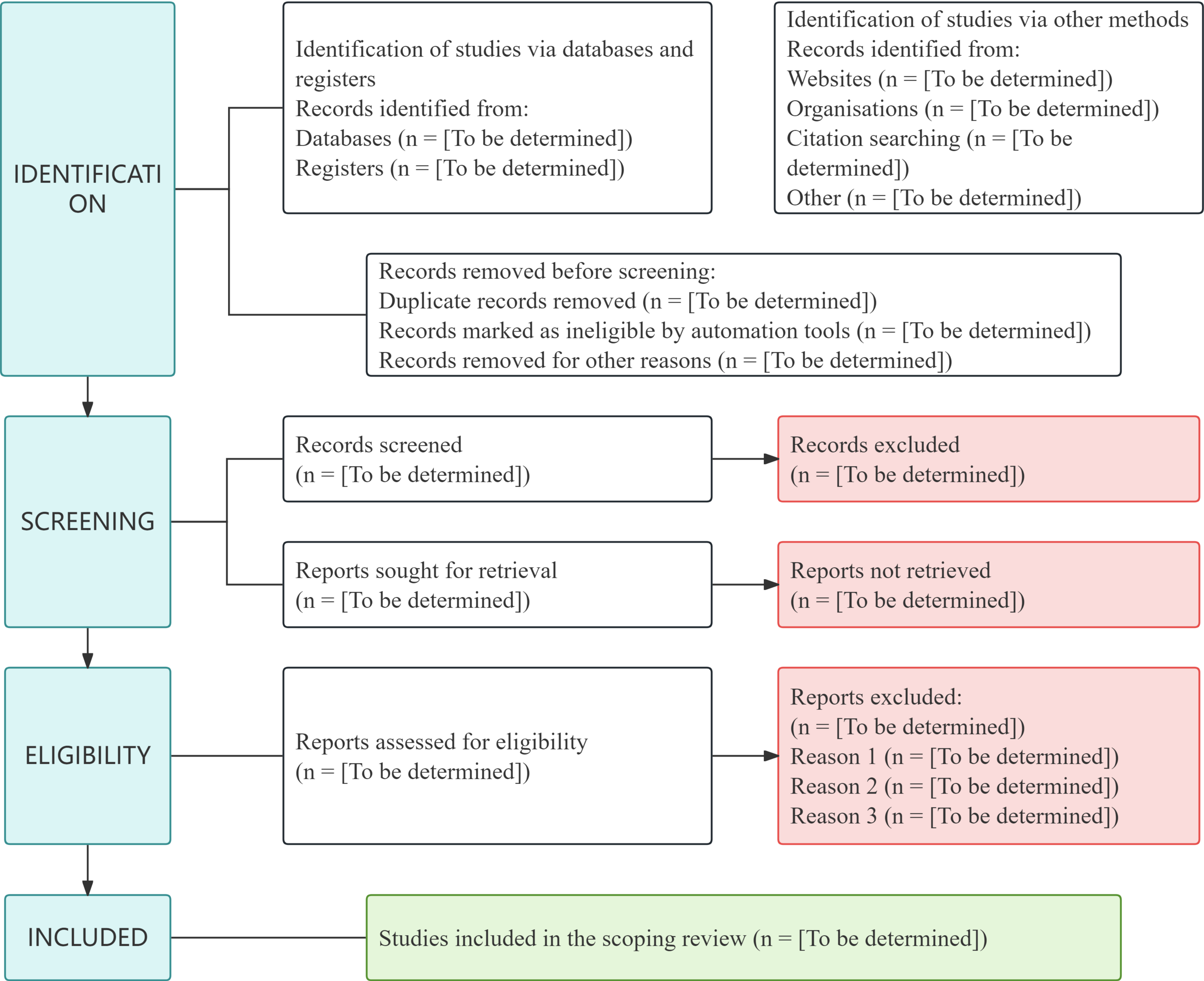

Supplement: S1 Fig — (PDF) [file pone.0329861.s003.pdf]
