# Supplementary material for: Remimazolam for anesthesia and sedation in pediatric ambulatory surgery: A scoping review protocol
Source: PLoS One. 2025 Aug 5;20(8):e0329861. doi: 10.1371/journal.pone.0329861 (PMC12324083; doi:10.1371/journal.pone.0329861)
Supplement: S2 Checklist — (DOCX) [file pone.0329861.s004.docx]

| **Section and Topic** | **Item #** | **Checklist item** | **Location where item is reported** |
| --- | --- | --- | --- |
| **TITLE** | | |  |
| Title | 1 | Identify the report as a systematic review. | Title (explicitly states “A Scoping Review Protocol”) |
| **ABSTRACT** | | |  |
| Abstract | 2 | See the PRISMA 2020 for Abstracts checklist. | Abstract section |
| **INTRODUCTION** | | |  |
| Rationale | 3 | Describe the rationale for the review in the context of existing knowledge. | Introduction (Rationale subsection) |
| Objectives | 4 | Provide an explicit statement of the objective(s) or question(s) the review addresses. | Introduction (Objectives subsection and subsequent list of essential questions) |
| **METHODS** | | |  |
| Eligibility criteria | 5 | Specify the inclusion and exclusion criteria for the review and how studies were grouped for the syntheses. | Methods (Inclusion Criteria subsection including Population, Concept, Context; Study Types subsection) |
| Information sources | 6 | Specify all databases, registers, websites, organisations, reference lists and other sources searched or consulted to identify studies. Specify the date when each source was last searched or consulted. | Methods (Information Sources subsection; Search Strategy subsection for search date) |
| Search strategy | 7 | Present the full search strategies for all databases, registers and websites, including any filters and limits used. | Methods (Search Strategy subsection), Appendix A |
| Selection process | 8 | Specify the methods used to decide whether a study met the inclusion criteria of the review, including how many reviewers screened each record and each report retrieved, whether they worked independently, and if applicable, details of automation tools used in the process. | Methods (Literature Screening subsection) |
| Data collection process | 9 | Specify the methods used to collect data from reports, including how many reviewers collected data from each report, whether they worked independently, any processes for obtaining or confirming data from study investigators, and if applicable, details of automation tools used in the process. | Methods (Data Extraction subsection) |
| Data items | 10a | List and define all outcomes for which data were sought. Specify whether all results that were compatible with each outcome domain in each study were sought (e.g. for all measures, time points, analyses), and if not, the methods used to decide which results to collect. | Methods (Data Extraction subsection - refers to Table 1), Table 1 (Outcome Measures & Main Findings section) |
|  | 10b | List and define all other variables for which data were sought (e.g. participant and intervention characteristics, funding sources). Describe any assumptions made about any missing or unclear information. | Methods (Data Extraction subsection - refers to Table 1), Table 1 (Categories: Study Characteristics, Participant Characteristics, Intervention Details, etc.) |
| Study risk of bias assessment | 11 | Specify the methods used to assess risk of bias in the included studies, including details of the tool(s) used, how many reviewers assessed each study and whether they worked independently, and if applicable, details of automation tools used in the process. | Methods (Data Synthesis and Presentation of Results subsection - explicitly states risk of bias assessment is not intended) |
| Effect measures | 12 | Specify for each outcome the effect measure(s) (e.g. risk ratio, mean difference) used in the synthesis or presentation of results. | Methods (Data Synthesis and Presentation of Results subsection - states meta-analysis for effect estimates is not intended) |
| Synthesis methods | 13a | Describe the processes used to decide which studies were eligible for each synthesis (e.g. tabulating the study intervention characteristics and comparing against the planned groups for each synthesis (item #5)). | Methods (Data Synthesis and Presentation of Results subsection - describes narrative summary organized by research questions) |
|  | 13b | Describe any methods required to prepare the data for presentation or synthesis, such as handling of missing summary statistics, or data conversions. | Methods (Data Synthesis and Presentation of Results subsection - narrative summary, tables, figures) |
|  | 13c | Describe any methods used to tabulate or visually display results of individual studies and syntheses. | Methods (Data Synthesis and Presentation of Results subsection) |
|  | 13d | Describe any methods used to synthesize results and provide a rationale for the choice(s). If meta-analysis was performed, describe the model(s), method(s) to identify the presence and extent of statistical heterogeneity, and software package(s) used. | Methods (Data Synthesis and Presentation of Results subsection - narrative summary, no meta-analysis) |
|  | 13e | Describe any methods used to explore possible causes of heterogeneity among study results (e.g. subgroup analysis, meta-regression). | Not applicable (as per 13d, no meta-analysis planned, thus no formal heterogeneity exploration) |
|  | 13f | Describe any sensitivity analyses conducted to assess robustness of the synthesized results. | Not applicable (as per 13d, no meta-analysis planned, thus no sensitivity analyses) |
| Reporting bias assessment | 14 | Describe any methods used to assess risk of bias due to missing results in a synthesis (arising from reporting biases). | Not explicitly stated for formal assessment in synthesis; Methods (Information Sources - implies attempt to find unpublished studies) |
| Certainty assessment | 15 | Describe any methods used to assess certainty (or confidence) in the body of evidence for an outcome. | Discussion (Preliminary Expectations subsection - states no comprehensive quality assessment, implying no formal certainty assessment) |
| **RESULTS** | | |  |
| Study selection | 16a | Describe the results of the search and selection process, from the number of records identified in the search to the number of studies included in the review, ideally using a flow diagram. | Methods (Literature Screening subsection - states PRISMA-ScR flow chart will be used) |
|  | 16b | Cite studies that might appear to meet the inclusion criteria, but which were excluded, and explain why they were excluded. | Methods (Literature Screening subsection - states rationale for exclusion will be noted) |
| Study characteristics | 17 | Cite each included study and present its characteristics. | Methods (Data Extraction subsection; Data Synthesis and Presentation of Results subsection) |
| Risk of bias in studies | 18 | Present assessments of risk of bias for each included study. | Methods (Data Synthesis and Presentation of Results subsection - states not intended) |
| Results of individual studies | 19 | For all outcomes, present, for each study: (a) summary statistics for each group (where appropriate) and (b) an effect estimate and its precision (e.g. confidence/credible interval), ideally using structured tables or plots. | Methods (Data Extraction subsection; Data Synthesis and Presentation of Results subsection - narrative summary and tables) |
| Results of syntheses | 20a | For each synthesis, briefly summarise the characteristics and risk of bias among contributing studies. | Methods (Data Synthesis and Presentation of Results subsection - refers to narrative summary) |
|  | 20b | Present results of all statistical syntheses conducted. If meta-analysis was done, present for each the summary estimate and its precision (e.g. confidence/credible interval) and measures of statistical heterogeneity. If comparing groups, describe the direction of the effect. | Methods (Data Synthesis and Presentation of Results subsection - no meta-analysis planned) |
|  | 20c | Present results of all investigations of possible causes of heterogeneity among study results. | Not applicable (no meta-analysis planned) |
|  | 20d | Present results of all sensitivity analyses conducted to assess the robustness of the synthesized results. | Not applicable (no meta-analysis planned) |
| Reporting biases | 21 | Present assessments of risk of bias due to missing results (arising from reporting biases) for each synthesis assessed. | Not applicable for synthesis assessment (as per item 14 for a scoping review) |
| Certainty of evidence | 22 | Present assessments of certainty (or confidence) in the body of evidence for each outcome assessed. | Not applicable for formal assessment (as per item 15 for a scoping review) |
| **DISCUSSION** | | |  |
| Discussion | 23a | Provide a general interpretation of the results in the context of other evidence. | Discussion (Preliminary Expectations subsection); Expected Results and Dissemination section |
|  | 23b | Discuss any limitations of the evidence included in the review. | Discussion (Preliminary Expectations subsection) |
|  | 23c | Discuss any limitations of the review processes used. | Discussion (Preliminary Expectations subsection) |
|  | 23d | Discuss implications of the results for practice, policy, and future research. | Objective (point 4); Expected Results and Dissemination section; Conclusion section; Discussion (Preliminary Expectations subsection) |
| **OTHER INFORMATION** | | |  |
| Registration and protocol | 24a | Provide registration information for the review, including register name and registration number, or state that the review was not registered. | Not stated in this protocol manuscript (manuscript itself is the protocol, registration status not mentioned) |
|  | 24b | Indicate where the review protocol can be accessed, or state that a protocol was not prepared. | This document/manuscript itself. |
|  | 24c | Describe and explain any amendments to information provided at registration or in the protocol. | Not applicable at protocol stage. |
| Support | 25 | Describe sources of financial or non-financial support for the review, and the role of the funders or sponsors in the review. | Funding section |
| Competing interests | 26 | Declare any competing interests of review authors. | Declaration of Competing Interest section |
| Availability of data, code and other materials | 27 | Report which of the following are publicly available and where they can be found: template data collection forms; data extracted from included studies; data used for all analyses; analytic code; any other materials used in the review. | Table 1 (Data Extraction Form provided in the initial user prompt, referenced in protocol); Availability of other future materials not explicitly stated. |

*From:*  Page MJ, McKenzie JE, Bossuyt PM, Boutron I, Hoffmann TC, Mulrow CD, et al. The PRISMA 2020 statement: an updated guideline for reporting systematic reviews. BMJ 2021;372:n71. doi: 10.1136/bmj.n71. This work is licensed under CC BY 4.0. To view a copy of this license, visit <https://creativecommons.org/licenses/by/4.0/>
