# Supplementary material for: Remimazolam for anesthesia and sedation in pediatric ambulatory surgery: A scoping review protocol
Source: PLoS One. 2025 Aug 5;20(8):e0329861. doi: 10.1371/journal.pone.0329861 (PMC12324083; doi:10.1371/journal.pone.0329861)
Supplement: Attachment — Submitted filename: Response to Reviewers.docx [file pone.0329861.s006.docx]

Dear Dr. Kiyan Heybati, Academic Editor, and Distinguished Reviewers:

We are deeply grateful for your thorough and constructive review of our manuscript "Remimazolam for Anesthesia and Sedation in Pediatric Ambulatory Surgery: A Scoping Review Protocol" (PONE-D-25-27249). Your insightful comments have been invaluable in strengthening our protocol, and we have carefully addressed each point raised. We believe these revisions have substantially improved the clarity, rigor, and potential impact of our work.

# Response to Editorial Requirements:

## 1. PLOS ONE Style Requirements

We sincerely apologize for not adhering to the journal's formatting standards in our initial submission. We have now meticulously reformatted the entire manuscript according to PLOS ONE templates, including proper file naming conventions. We appreciate your patience and have ensured full compliance with all style requirements.

## 2. Funding Disclosure Statement

Thank you for highlighting this important omission. We have added the following statement to our cover letter and manuscript: "The funders had no role in study design, data collection and analysis, decision to publish, or preparation of the manuscript." This accurately reflects the complete independence of our research from funding influence.

## 3. Table 1 Inclusion

We apologize for this oversight. Table 1 has now been fully integrated into the manuscript (page 7), providing a comprehensive data extraction framework that clearly delineates all variables to be collected during our review process.

# Response to Reviewer 1:

We greatly appreciate your positive assessment of our protocol's potential contribution to understanding remimazolam use in pediatric populations.

## 1. Data Availability Statement

We apologize for omitting this crucial information. We have now added a clear data availability statement: "All data extracted during this scoping review will be made publicly available upon study completion through an open-access repository (such as Open Science Framework or Figshare)." This commitment ensures transparency and facilitates future research building upon our findings.

# Response to Reviewer 2:

We are extremely grateful for your detailed and thoughtful review, which has significantly enhanced our manuscript's quality.

## 1. Background Section Length and Repetition

Your observation was absolutely correct. We have substantially revised the background section, reducing it by approximately 40% while maintaining all essential information. Specifically:

Consolidated repetitive statements about pharmacokinetic properties into a single, comprehensive sentence

Removed redundant safety profile descriptions

Streamlined the rationale into three focused paragraphs that flow logically from general context to specific research gaps

## 2. Objectives Reorganization

Following your excellent suggestion, we have transformed our objectives into seven clearly numbered research questions. This format dramatically improves readability and allows readers to quickly identify our specific aims. Each question now addresses a distinct aspect of remimazolam use in pediatric ambulatory surgery.

## 3. Google Scholar Results Justification

Thank you for requesting this clarification. We have added the following rationale: "first 200-300 results, as these typically contain the most relevant citations based on relevance ranking algorithms, with diminishing returns beyond this threshold based on empirical testing in previous scoping reviews."

## 4. Dual Software Usage Clarification

We have clarified our software selection: "EndNote serves as our reference management system for citation organization and automated deduplication, while Rayyan provides superior collaborative features for the screening process, allowing real-time coordination between reviewers with conflict resolution capabilities."

## 5. Language and Style Improvements

We have comprehensively revised the manuscript's language:

Simplified complex sentences (average sentence length reduced from 28 to 19 words)

Converted passive constructions to active voice where appropriate (approximately 70% of previously passive sentences)

Corrected all typographical errors identified

Standardized terminology throughout (e.g., consistently using "ambulatory surgery" rather than alternating with "outpatient surgery")

# Response to Reviewer 3:

We deeply appreciate your comprehensive feedback, which has prompted substantial enhancements to our protocol. We have carefully addressed all seven points you raised, organizing our responses into five integrated sections that comprehensively cover all your concerns:

## 1. Table Format Summary (Addressing your points 1 & 2)

Thank you for emphasizing that this is a scoping review and requesting a table format summary. Table 1 is now included on page 7, presenting a structured framework for data extraction that will facilitate systematic and reproducible data collection. This table clearly demonstrates our scoping review approach by outlining all variables to be collected without quality assessment metrics.

## 2. Factors Affecting Study Outcomes (Addressing your point 3)

We have added a dedicated subsection in the Discussion titled "Factors Affecting Study Outcomes" that addresses factors influencing heterogeneity:

Study design variations and their impact on outcome reliability

Population characteristics (age distribution, comorbidity profiles)

Geographic and healthcare system differences

Measurement standardization challenges

This analysis will help readers interpret our findings within appropriate context.

## 3. Expanded Discussion and Limitations (Addressing your points 4 & 5)

The Discussion section has been substantially expanded from 450 to 1,200 words, now including:

Detailed analysis of anticipated findings across different pediatric age groups

Comprehensive exploration of potential clinical implications

Systematic identification of research priorities

A dedicated "Study Limitations" subsection that explicitly acknowledges methodological limitations inherent to scoping reviews

Discussion of potential language bias and strategies to minimize its impact

## 4. Novel Knowledge Contribution (Addressing your point 6)

We have explicitly stated our unique contribution in the "Novel Contributions" subsection: "This represents the first systematic evidence synthesis specifically examining remimazolam use in pediatric ambulatory surgery, filling a critical gap between expanding adult literature and limited pediatric experience. Our comprehensive mapping will provide clinicians with an evidence-based foundation for practice decisions while highlighting priority areas for future research."

## 5. Clinical Practice Applications (Addressing your point 7)

A new subsection "Clinical Practice Implications" provides specific guidance on how to apply this knowledge:

Decision-making framework for patient selection based on available evidence

Risk stratification approach for different age groups and comorbidities

Practical considerations for dosing strategy selection

Clear identification of clinical scenarios requiring additional caution

These practical applications directly address how readers can apply our findings in clinical practice, supporting evidence-based decision-making in pediatric ambulatory settings.

# PLOS ONE Formatting

All formatting has been standardized according to journal requirements, with particular attention to reference formatting, figure labeling, and supplementary material organization.

Summary of Major Improvements:

Structural Enhancement: Streamlined background, clarified objectives, expanded discussion

Methodological Clarity: Detailed software rationale, explicit inclusion/exclusion criteria, comprehensive data extraction framework

Clinical Relevance: Added practical implications section, addressing real-world application needs

Transparency: Clear data sharing commitment, acknowledged limitations, funding independence statement

Language Quality: Improved readability, consistency, and academic precision throughout

We believe these comprehensive revisions address all reviewer concerns while substantially strengthening our protocol's scientific rigor and practical utility. We are confident that this scoping review will make a meaningful contribution to pediatric anesthesia practice and research.

Thank you again for your valuable feedback and for considering our revised manuscript. We look forward to your response and remain available to address any additional concerns.

Sincerely,

Yunbo Mo

Corresponding Author

On behalf of all authors
